# It’s not *what* you do, it’s the *way* that you do it: An experimental task delineates among passive, reactive and interactive styles of behaviour on social networking sites

**DOI:** 10.1371/journal.pone.0276765

**Published:** 2022-12-07

**Authors:** Daniel J. Shaw, Linda K. Kaye, Nicola Ngombe, Klaus Kessler, Charlotte R. Pennington

**Affiliations:** 1 School of Psychology, College of Health & Life Sciences, Aston University, Birmingham, United Kingdom; 2 Institute of Health & Neurodevelopment, Aston University, Birmingham, United Kingdom; 3 Department of Psychology, Edge Hill University, Ormskirk, United Kingdom; 4 Institute of Clinical Psychology and Psychotherapy, Technische Universität Dresden, Dresden, Germany; 5 School of Psychology, University College Dublin, Dublin, Ireland; University of Exeter, UNITED KINGDOM

## Abstract

Studies have produced vastly disparate findings when exploring relationships between social networking site (SNS) usage and psychosocial well-being. These inconsistencies might reflect a lack of consideration for *how* people use SNS; specifically, while meaningful interactions are suggested to foster positive feelings, the passive consumption of others’ feeds is proposed to have negative effects on users’ well-being. To facilitate the empirical evaluation of these claims, the present study developed a computerised task to measure styles of usage on a mock SNS platform. Administering this Social Network Site Behaviour Task (SNSBT) online to 526 individuals, we identified three dissociable usage styles that extend the active-passive dichotomy employed frequently in the literature: passive use (consuming content posted by others), *re*active use (reacting to others’ content), and *inter*active use (interacting with others through content sharing). Furthermore, our data reveal that these usage styles differ on several measures of psychosocial variables employed frequently in the disparate literature: more interactive users reported greater feelings of social connectedness and social capital than passive or reactive users. Importantly, however, our results also reveal the multi-dimensional nature of usage styles, with online network size and time spent on SNS platforms serving as potentially confounding influences on some psychosocial measures. These findings not only advance our understanding of SNS behaviour by providing empirical support for theoretic propositions, but also demonstrate the utility of the SNSBT for experimental investigations into the psychosocial outcomes of different SNS usage styles.

## Introduction

Social networking sites (SNS; e.g., Facebook, Instagram) have become a ubiquitous feature of our daily lives; an estimated 3.6 billion people worldwide used them daily in 2020, and this is predicted to rise to 4.4 billion in 2025 [1]. By providing channels that allow people to connect socially with vast networks, many scholars consider the increased usage of SNS, and social media platforms more generally, to serve important psychosocial benefits. Nevertheless, concerns are also raised over the negative effects on our subjective well-being from SNS replacing meaningful ‘real-world’ interactions (see [2–4]). This debate is fuelled partly by discrepancies in research findings, which might reflect a lack of consideration for differences in the way that people engage with SNS [5]. To facilitate the reconciliation of these inconsistencies, the present study developed a computerised task to measure styles of behaviour on a mock SNS–one that can be used in future research to assess empirically the psychosocial outcomes of dissociable styles of usage. To assess the potential utility of the task for this means, we examined whether distinct usage styles differ on several psychosocial variables evaluated commonly as outcome measures in this literature: loneliness, sense of belonging, social connectedness and social capital.

Despite a wealth of research into the psychological impact of increasing SNS usage, inconsistent findings have prevented any firm conclusions being drawn. A full review of this burgeoning literature is beyond the scope of this paper, and readers are advised to consult more comprehensive overviews provided elsewhere (e.g., [2, 3, 5–8]). In brief, while a plethora of studies report that social media usage is associated with increased social connectedness and reduced loneliness [9–12], others have observed detriments to loneliness and well-being from greater use of such platforms (e.g., [13–15]) or report no meaningful relationships between social media use and indices of psychological well-being (e.g., [16–19]).

According to the interpersonal-connection-behaviours framework [20], such disparities likely reflect differences in styles of social media usage: while directed communication, posting, and sharing of content (active usage) are believed to enhance users’ psychological well-being by increasing social connectedness and decreasing loneliness [21], non-communicative consumption of feeds (passive usage) is said to increase experiences of isolation and decrease self-esteem (e.g., [22, 23]; for a review see [24]). This highlights the need for accurate measurements of user behaviour on SNS if we are to understand the psychosocial outcomes associated with increasing usage ([8, 25]; see also [26]).

A recent meta-analysis has revealed the vast number of self-report instruments that have been developed for measuring styles of SNS usage along the active-passive dichotomy [25]. Recently, however, many scholars have questioned the usefulness of this crude categorisation, calling for a more nuanced refinement (e.g., [5]). It is also argued that the field of cyberpsychology needs to move beyond subjective self-report questionnaires and consider behavioural methods that allow for more direct assessments of usage styles on SNS [18]. One alternative method is to use digital tracking with data recorded directly from devices [27–30]. Importantly, though, while such direct tracking of activity is considered widely to be the gold standard method (see [17, 31]), the necessary reliance upon the willingness of individuals to allow researchers to monitor their (natural) usage raises questions concerning sampling bias [32]. Further, tracking measurements of SNS usage will vary markedly across software platforms and measurement periods (e.g., weekday *vs*. weekend) and become inaccurate when individuals have access to such sites through multiple unlinked devices [32].

Assessing social media usage objectively whilst avoiding these limitations is achievable with a behavioural measure that can be administered under controlled experimental conditions. To capture different styles of usage that might relate differentially to user outcomes, such a measure must go beyond unidimensional assessments of engagement (e.g., number of log-ins, activity duration) and delineate among individuals who exhibit different behavioural patterns [25]. As reviewed above, evidence suggests that active engagement with others on SNS is more likely to be associated with positive psychosocial measures when compared with passive use. More recent research suggests that usage styles can be divided further, however; a recent meta-analysis distinguished between one-sided (e.g., liking) and bidirectional active social exchanges (commenting) in computer-mediated communication [33]. Similarly, other research has delineated between public (e.g., bidirectional chatting) and private (e.g., tagging) active engagement [8]. If the degree to which individuals engage in meaningful *inter*actions with other users is indeed a key factor in the positive outcomes of active SNS usage, as proposed by the interpersonal-connection-behaviours framework [20], a useful behavioural measure should be capable of dissociating empirically among these passive, reactive (unidirectional) and interactive (bidirectional) forms of usage.

To investigate if these more nuanced styles of usage can be identified behaviourally, the present study developed the Social Networking Site Behaviour Task (SNSBT)–a mock SNS platform based loosely on Facebook and Instagram. As a means of validating this task, we then examined if dissociable patterns of reactive and interactive behaviour converged with subjective perceptions of usage style assessed with a commonly used self-report instrument based on the active-passive dichotomy [34], which we modified to distinguish further between reactive and interactive usage. Finally, we examined whether distinct usage styles identified on this mock SNS differed in terms of several psychosocial factors employed frequently in the disparate literature. Importantly, this was not to assess the outcomes of dissociable styles, which requires pre- and post-usage comparisons or real-time measurements (e.g., [13]); rather, this evaluated the potential utility of the SNSBT for future research and reconciling inconsistent findings. First we assessed differences in self-reported *loneliness* and *sense of belonging*, the latter referring to the degree to which an individual values their involvement in, or feels valued by others within a social system [35]. Since social interactions are important for satisfying socioemotional needs, including the need for belonging (see [20]), we predicted that greater interactive usage on the SNSBT would be associated with less loneliness and an increased sense of belonging relative to more reactive and passive usage. Similarly, we investigated relationships between patterns of usage on the mock SNS and self-reported *social connectedness*–the experience of belonging to a social network or community [36], and *social capital*–the perceived outcomes (e.g., emotional support) that individuals take from online relationships with close friends and family (bonding) or more extended networks (bridging; [37]). Given the emerging evidence for opposing associations between self-reported active or passive usage and these psychosocial outcomes (see [24, 33]), we predicted that subjective reports of social connectedness and social capital would be greater from individuals showing more interactive usage on the SNSBT compared with reactive or passive usage. Finally, as an exploratory analysis, we investigated whether dissociable styles of behaviour on our experimental task differed on other subjective measures of SNS usage shown previously to relate to psychosocial variables—namely, the size of one’s online social network [38, 39] and the number of hours spent on SNS platforms [40].

## Method

### Participants

Participants were recruited through Prolific Academic (www.prolific.co), which has been shown to avoid some of the shortcomings associated with other crowdsourcing platforms [41]. To be eligible for participation, individuals had to be (1) over the age of 18, (2) a Facebook user, and (3) able to complete the study on a PC or laptop with a standard QWERTY keyboard in a single continuous session. Participation was recompensed at £7.50/hour. In total, 622 Prolific identifiers were initially recruited, a sample size determined solely by available funding. Of these, 25 individuals were removed because they had participated more than once. Of the remaining 596, we removed 69 who provided incomplete data across measures (see below) and two who reported no prior Facebook use. The final sample therefore comprised 526 participants (*M*_*AGE*_ = 31.07 years, *SD* = 11.41, range = 18–71; 254 males). Sensitivity power analyses were conducted using the *pwr* package in R-Studio [42]; based on the cluster analyses described below, these indicated that we were able to detect effects of Cohen’s *d* > .28 with 80% power at *α* = .05.

All volunteers provided written informed consent and the study was approved by the University Research Ethics Committee at Aston University (ref. 1649).

### Procedure

Participants completed a large battery of measures comprising the Social Networking Site Behaviour Task and five self-report instruments. The former was administered through Pavlovia (https://pavlovia.org)–an online platform for running PsychoPy experiments (v3; [43]). Upon completion of this task, participants were re-directed automatically to Qualtrics (www.qualtrics.com) where they completed the five questionnaires. The SNSBT task was always completed first to avoid any priming from explicit questionnaire-based content, and the subsequent self-report instruments were presented randomly to minimise order effects. The entire experiment lasted approximately 45 minutes. To ensure data integrity, an attention check was embedded among the questionnaires (“How many months are there in a year?”) that required participants to select one of four possible answers. All participants answered this check correctly.

### Measures

#### Social Networking Site Behaviour Task (SNSBT)

We developed the SNSBT to assess whether passive, reactive, and interactive behavioural tendencies could be identified on a mock SNS. Before the task, participants were informed that they would be connected to a network of 99 other ‘friends’ on a new social networking platform and would see a series of images posted by other members of that network. In response to each image, they were asked to either “Like” or “Share” it, or skip to the “Next” image. Participants were also told that, since not all members of this network were ‘friends’ with every other member, the images they chose to share would be seen for the first time by some people who might then comment on the shared post. Finally, to ensure their behaviour was representative of their typical SNS behaviour, participants received the following instruction:

“*Please take a moment to think about how you typically respond on social media (e*.*g*., *Facebook)*. *Do you “like” a lot of pictures*, *do you often “share” them*, *or do you usually scroll through images without liking or sharing them*? *We want you to act in the same way on this task as you would do on other social media platforms”*.

The images comprised 90 pictures of natural landscapes selected from the Nencki Affective Picture System (NAPS; [44, 45])–a validated and standardised set of high-quality photographs. Non-social images were selected purposefully to ensure that (un)familiarity of agents within the scenes could not influence responses. We used normative ratings provided for the NAPS to ensure that selected images were of neutral-positive valence (*M* = 7.12, *SD* = 0.74; range = 5.09–8.54) and low arousal (*M* = 3.59, *SD* = 0.67; range = 2.04–4.90). These normative ratings did not differ significantly between females and males (*p* > .05).

**Fig 1:**
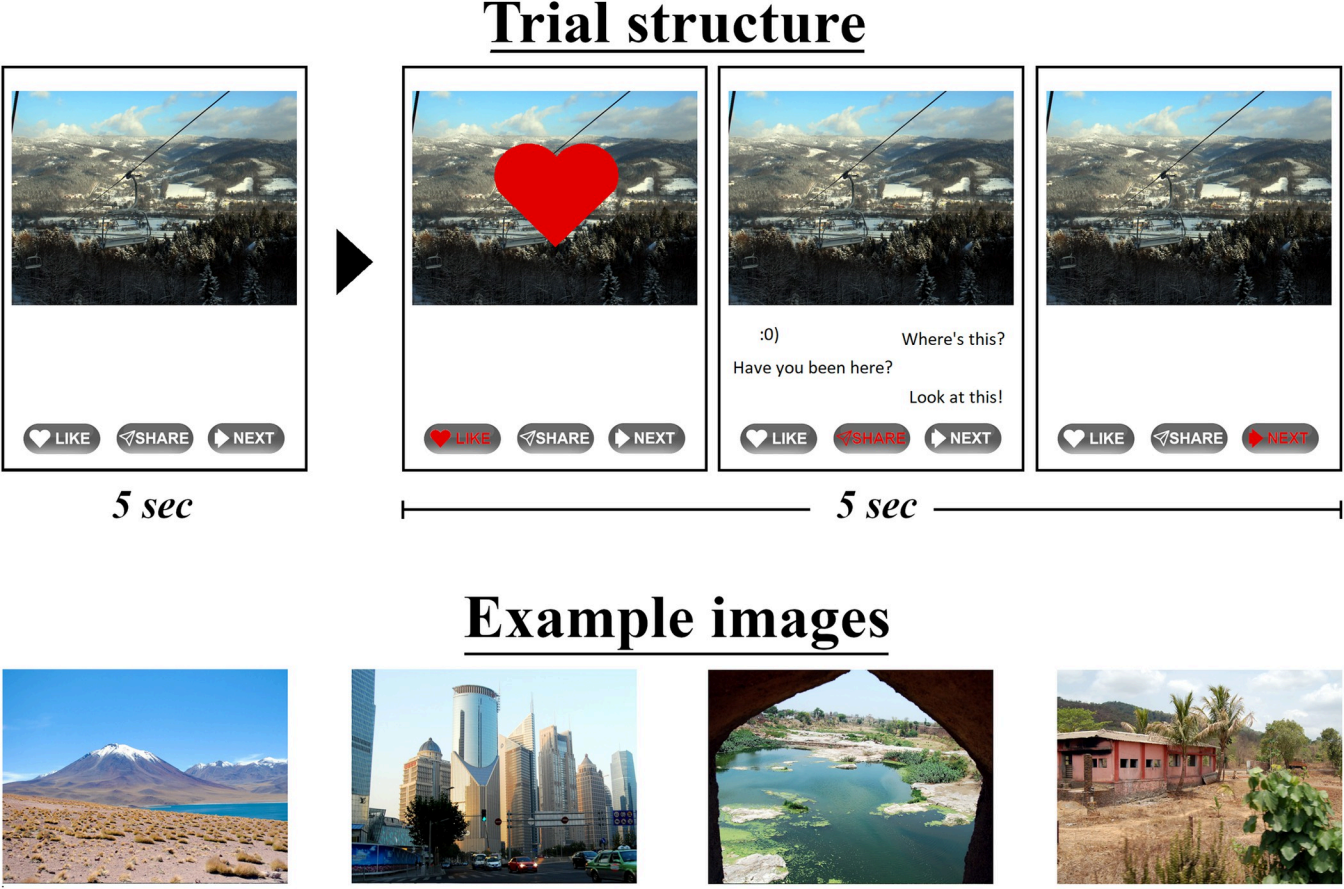
The Social Networking Site Behaviour Task (SNSBT). The top row illustrates the trial structure and three possible response options. The bottom row provides example images selected from the Nencki Affective Picture System. The example images are reprinted from the Nencki Affective Picture System [44] under a CC BY licence, with permission from Artur Marchewka, original copyright 2014.

Images were presented in a random sequence for a total of 90 trials. Participants were given 5 seconds to view and respond to each image by choosing “Like”, “Share” or “Next” (key 1, 2, or 3 on a standard computer, respectively). Upon a response, their selection was highlighted for a subsequent 5 seconds before the subsequent image appeared. When participants chose to “Like” an image, a symbol of a heart was presented to indicate that their reaction had been posted to the network. After choosing to “Share” an image, participants received immediate feedback in the form of three comments presented in short succession that were provided ostensibly by other members of the network. These comments were selected from publicly available Facebook content and matched meaningfully to each image (e.g., “*Have you been here*?”, “*I want to take my family here*”, “*I love this place*!”). When participants chose “Next”, the next image was displayed. Fig 1 depicts the trial structure. Responses to the images were shown to have excellent internal reliability (*a* = .96). For each participant, we calculated the proportion of “Like”, “Share” and “Next” responses across all 90 trials.

#### Self-reported social media usage

As a subjective measure of behaviour on social networking platforms, we adapted an existing self-report instrument that has been shown previously to dissociate between active and passive social media use (SMU; [34]). Importantly, the original seven-item version of this instrument does not differentiate between *re*- and *inter-*active styles; the four items that ask about liking, commenting, and sharing tendencies are subsumed under “active” usage. To assess whether these more nuanced styles of active SMU can be detected in subjective reports, we added two items such that passive, reactive, and interactive styles were each assessed with three distinct items (see Qualtrics Questionnaires; https://osf.io/z3ac6). Further, to avoid a potential ceiling effect from the temporal judgments used previously as response options (e.g., “*Several times a day*”), we used labels of frequency on a 5-point Likert scale (1 = *Never*, 5 = *Very Often*).

To assess the factor structure of this modified instrument, we performed Principal Component Analysis (PCA) with varimax rotation. Contrary to our expectations of a three-factor structure, this revealed an optimal two-factor solution (determinant of correlation matrix = .05; Kaiser-Meyer-Olkin = .81) replicating the original findings [34]. Six items measuring reactive and interactive behaviours accounted for 31.66% of variance and the remaining three assessing passive usage explained 25.66% (see S1 File). In light of these factor loadings, we label these as “Active” and “Passive” sub-scales. Both sub-scales demonstrated acceptable internal reliability (*α* = .80 and .75, respectively). Next, we computed a single index that reflected an individual’s self-reported tendency for active relative to passive SMU. Specifically, we calculated the difference between their mean rating across all items loading onto the Active factor and those loading onto the Passive factor; greater scores on this index of usage (SMU_*diff*_) indicate more active relative to passive usage. This relative measure was chosen to account for prior research findings; namely, studies have shown that while all users of social media engage most frequently in passive behaviours, they differ in their *relative* active engagement [46]. Indeed, the mean value of this difference measure across the sample was -.94 (*SD* = .83; range -3.82–1.67) indicating that participants reported more passive than active SNS usage.

#### Loneliness

Loneliness was measured using the eight-item UCLA Loneliness Scale-Short Form [47]. Participants respond to each item (e.g., “There is no one I can turn to”) on a 4-point Likert scale anchored between 1 (*Never*) and 4 (*Always*). This measure had good internal consistency (*α* = .87) and a total score across all items was computed, with higher scores representing greater feelings of loneliness. The mean across the sample was 17.09 (*SD* = 5.26; range 8–32).

#### Sense of belonging

Social belonging was measured using the Sense of Belonging Instrument (SOBI-P; [35]). This 18-item scale contains questions such as “I often wonder if there is any place on earth where I really fit in”, each of which is responded to on a 4-point Likert scale ranging between 1 (*Strongly Disagree*) and 4 (*Strongly Agree*). All items on the SOBI-P questionnaire are framed negatively, with higher scores representing lower social belonging. To aid interpretation alongside our other measures, we reverse scored the questionnaire so that higher scores represent greater sense of belonging. Responses were found to have excellent internal consistency (*α* = .95) and a total score was computed across items with higher scores repsenting greater social belonging. The mean across the present sample was 54.06 (*SD* = 13.21; range 19–72).

#### Social connectedness

Social connectedness was measured using the Social Connectedness Scale [36]. This questionnaire comprises 20 items (e.g., “I feel close to people”), and responses are provided via a 6-point Likert scale ranging from 1 (*Strongly Disagree*) to 6 (*Strongly Agree*). This measure resulted in excellent internal reliability (*α* = .95) and a total score was computed across items, with higher scores representing greater feelings of social connectedness. The mean score for the present sample was 80.75 (*SD* = 19.59; range 23–120).

#### Online social capital

Online social capital was measured using the Internet Social Capital Scale [37]. The original instrument comprises 40 items that ask individuals about both their online and offline social bonding and bridging experiences, but we focus exclusively on the online subscales that together comprise 20 items (10 items for bonding, 10 for bridging). For the current study, these subscale items were adapted to be conceptually correspondent with Facebook use (e.g., “When I feel lonely, there are several people on Facebook I can talk to”). Responses were made on a 5-point Likert scale ranging from 1 (*Not characteristic of me*) to 5 (*Extremely characteristic of me*). Both the bonding (*α* = .90) and bridging (*α* = .91) sub-scale resulted in excellent internal reliability. A total score was computed across items representing each subscale, with higher scores representing greater online social capital. Across the sample, the mean scores for the bonding and bridging subscale were 29.91 (*SD* = 9.44; range 10–50) and 31.10 (*SD* = 9.35; range 8–50), respectively.

### Exploratory measures

#### Online social network size

In addition to the aforementioned confirmatory measures which allowed us to evaluate our hypotheses directly, the online protocol also included three questions employed in another study [38] to assess whether styles of behaviour on the SNSBT were associated with the size of participants’ online social networks. Participants first responded to the questions “How many Facebook friends do you have?” and “How many friends do you have in your offline social circle?”. They then responded to the question “What percentage of Facebook friends do you consider to be genuine friends?”, but we do not consider answers to this question in the analyses that follow. Higher scores represent greater social networking size. The average number of Facebook friends was 415.38 (*SD* = 448.58), of whom 27.09% (*SD* = 25.05) were considered as genuine friends. Participants also reported an average of 27.40 (*SD* = 59.07) offline friends. We used participants’ total number of online friends (*Friends*) as an exploratory covariate in our analyses.

#### Facebook hours

To assess whether styles on usage on the SNSBT were also associated with the number of hours spent on SNS platforms, we asked participants two questions that measured their Facebook usage on a typical weekday (“On average, how much do you use Facebook on a weekday [Monday-Friday]?”) and weekend (“On average, how much do you use Facebook on a weekend day [Saturday & Sunday]?”). Given that self-reported usage was much greater during the weekend than during the weekdays and provided greater variability, we chose to focus on this particular measure of usage frequency (*Hours*) as an exploratory covariate.

### Analytic strategy

To assess whether dissociable styles of behaviour could be detected on the SNSBT, we first conducted a two-step cluster analysis on participants’ response patterns; namely, the proportion of trials in which they made a “Like”, “Share” or “Next” response. This particular clustering technique is considered more objective than alternatives because it determines cluster membership based upon statistical measures of fit (e.g., Bayesian Information Criterion [BIC]) and is capable of handling large datasets [48]. In line with published recommendations [49], we used the BIC index to determine the model fit and the log-likelihood to assess the probability that each observation (participant) belongs to a cluster.

Having identified the optimal number of clusters, next we performed a series of ANOVAs to ascertain the response tendencies of constituent members on the SNSBT and compared this to their self-reported usage (SMU_*diff*_). We then examined whether dissociable styles of behaviour on the task emerging from the cluster analysis differed on any of the psychosocial measures. In follow-up exploratory analyses, we examined whether types of usage on the SNSBT differed based on the number of friends and the number of hours spent on Facebook reported by individuals comprising each cluster. Given that the former was recorded in an ordinal fashion, this was assessed with non-parametric tests. Where the clusters differed on *Friends* and *Hours*, they were entered as (dummy-coded) covariates in follow-up ANCOVA analyses to determine if discrete patterns of SNS usage differed on any of the psychosocial measures independent of these covariates.

## Results

All analyses were performed in SPSS (v.26). In the following sections, means are presented with standard deviations (*SD*) and the results of all pairwise comparisons are reported after Bonferroni correction (*p*_*corr*_). Effect sizes are reported as partial eta squared for main effects, and Cohen’s *d* for mean differences [50].

### Cluster analysis

**Fig 2:**
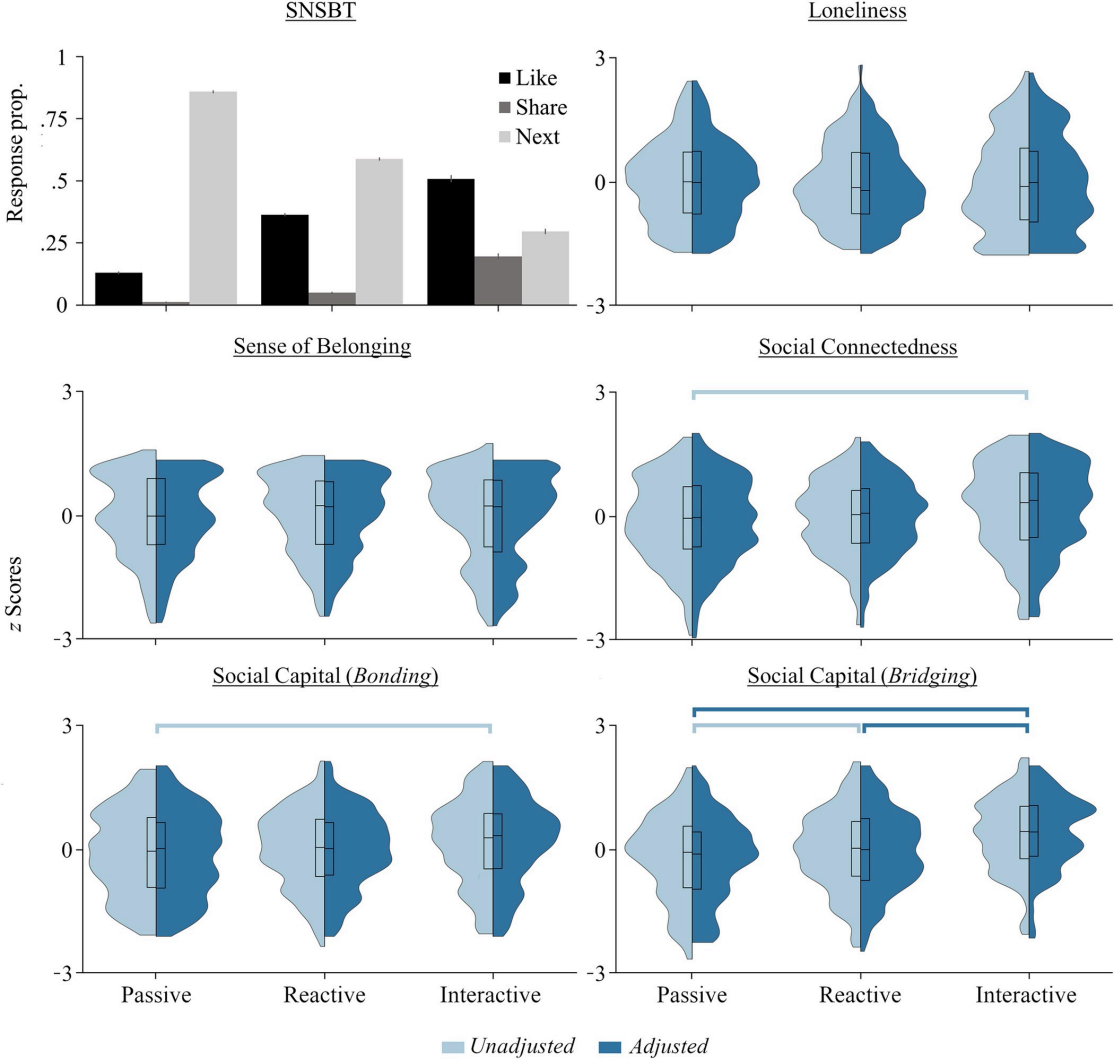
Behaviour on the experimental task and self-report ratings across the psychosocial variables for each cluster. Clockwise from top-left: Mean (±SE) proportions of the three possible response options on the SNSBT across individuals comprising each cluster; distributions of ratings within each cluster for the UCLA Loneliness Scale, Sense of Belonging Instrument, Social Connectedness Scale, and Internet Social Capital Scale. *Note*: Split violin plots present z-scored raw (unadjusted) and residualised ratings from the ANCOVA (adjusted for both the *Friends* and *Time* covariates). Plots are cut at minimum and maximum values, with inner boxplots presenting medians, first and third quartiles. C1 = cluster #1 (*Passive*), C2 = cluster #2 (*Reactive*), C3 = cluster #3 (*interactive*). Horizontal lines indicate significant differences between clusters for raw and adjusted ratings (light or dark blue, respectively).

The BIC change ratio indicated an optimal three-cluster solution. A Silhouette measure of cohesion and separation indicated good model fit (.60), and the ratio of distance measures was acceptable (2.09). Each cluster differed significantly in terms of the proportion of “Next” (*F*[2,523] = 1403.67, *p* < .001, *η*_*p*_^*2*^ = .84), “Like” (*F*[2,523] = 505.69, *p* < .001, *η*_*p*_^*2*^ = .66) and “Share” (*F*[2,523] = 246.09, *p* < .001, *η*_*p*_^*2*^ = .49) responses: Participants in Cluster 1 made the highest proportion of “Next” but the fewest “Like” and “Share” responses (*p*_*corr*_ < .001), while those in Cluster 3 made the least number of “Next” but the highest proportion of “Like” and “Share” responses (*p*_*corr*_ < .001). Those in Cluster 2 made an intermediate proportion of “Next”, “Share” and “Like” responses relative to the other two clusters (*p*_*corr*_ < .001). Based on these response patterns, we refer to Cluster 1 herein as *Passive*, Cluster 2 as *Reactive*, and Cluster 3 as *Interactive*. The response patterns of each cluster are illustrated in Fig 2.

### Cluster comparisons

A one-way ANOVA comparing the three clusters on the SMU_*diff*_ variable revealed that, while all participants considered themselves to exhibit stronger passive than active usage on Facebook (indicated by negative scores on this relative measure), these self-perceptions differed significantly between each cluster in line with their behaviour on the task (*F*[2,523] = 14.42, *p* < .001, *η*_*p*_^*2*^ = .05). Individuals in the *Passive* cluster produced the lowest score (-1.14 [.85]), indicating the strongest self-perceived passive tendency, while the *Interactive* cluster scored the highest (-.65 [.77]) and members of the *Reactive* cluster scored at an intermediate level (-.93 [.80], *p*_corr_ < .05).

When comparing the clusters on each of the psychosocial measures, a monotonically decreasing pattern emerged for the *Bridging* subscale of *Social Capital* (*F*[2,523] = 17.80, *p* < .001, *η*_*p*_^*2*^ = .06); the *Interactive* cluster scoring significantly higher (34.85 [8.35]) than the *Reactive* (30.82 [9.06]; *p* < .001, *d* = .46) and *Passive* clusters (28.88 [9.51]; *p* < .001, *d* = .67), and individuals in the *Reactive* cluster scoring significantly higher than their *Passive* counterparts (*p* = .035, *d* = .21). There was also a main effect of Cluster on the *Bonding* subscale (*F*[2,523] = 3.53, *p* = .030, *η*_*p*_^*2*^ = .01), whereby individuals in the *Interactive* cluster scored higher (31.61 [9.61]) than those in the *Passive* cluster (28.84 [9.77], *p* = .025, *d* = .29), but there were no differences between those in the *Reactive* cluster (29.85 [8.80]) and either the *Interactive* or *Passive* clusters (*p*>.296, *d* = .19 and .11, respectively). A similar pattern emerged for self-reported *Social Connectedness* (*F*[2,523] = 3.38, *p* = .035, *η*_*p*_^*2*^ = .01); those in the *Interactive* cluster scored significantly higher (84.29 [20.77]) than the *Passive* cluster (78.70 [20.13]; *p* = .030, *d* = .27), but there were no significant differences between the *Interactive* and *Reactive* (80.45 [17.77]; *p* = .246, *d* = .20) or the *Reactive* and *Passive* clusters (*p*>.999, *d* = .09). There was no significant difference between the clusters in terms of *Sense of Belonging* (*F*[2,523] = .153, *p* = .858, *η*_*p*_^*2*^ = .001) or *Loneliness* (*F*[2,523] = .174, *p* = .840, *η*_*p*_^*2*^ = .001).

An exploratory ANOVA revealed that the three clusters also differed significantly in the number of self-reported Facebook friends (*F*[2,523] = 4.96, *p* = .007, *η*_*p*_^*2*^ = .02): Individuals comprising the *Interactive* cluster reported significantly more *Friends* (486.65 [526.85]) than those the *Passive* cluster (341.58 [345.43], *p*_*corr*_ = .010, *d* = .33), while members of the *Reactive* cluster (444.98 [477.28]) did not differ significantly from their *Interactive* (*p*_*corr*_>.999, *d* = .08) or *Passive* counterparts (*p*_*corr*_ = .067, *d* = .25). Furthermore, a non-parametric Kruskall-Wallis test demonstrated that the clusters differed significantly in the ordinal number of weekend hours they spent on Facebook (H[2] = 35.01, *p* < .001, *ε*^*2*^ = .06]). Follow-up Mann-Whitney tests showed that the *Interactive* cluster reported spending more *Hours* on Facebook at the weekend (median = 2–3 hours) than the *Reactive* (median = 1–2 hours; *Z* = 2.56, *p* < .001) and *Passive* clusters (median = 1–2 hours; *Z* = 5.69, *p* < .001), and the *Reactive* cluster reported more *Hours* than the *Passive* users (*Z* = 3.70, *p* < .001). Given that the clusters differed in terms of both the *Friends* and *Hours*, these were added as covariates in exploratory ANCOVA analyses to assess their potentially confounding influence on the aforementioned differences between usage styles and psychosocial measures. The adjusted means estimated from this ANCOVA, together with other cluster characteristics, are presented in Table 1.

**Table 1:** Cluster characteristics across all measures.

	Cluster 1 (*Passive*)	Cluster 2 (*Reactive*)	Cluster 3 (*Interactive*)
N (%)	205 (39.0)	186 (35.4)	135 (25.7)
Males:Females	100:105	79:102	75:59
	Means (±*SD*)		
Age	31.30 (11.20)	30.65 (10.94)	31.32 (12.39)
Next	.86 (.08)	.59 (.08)	.30 (.13)
Like	.13 (.07)	.36 (.09)	.51 (.17)
Share	.01 (.02)	.05 (.05)	.20 (.14)
SMU_*diff*_	-1.14 (.85)	-.94 (.80)	-.65 (.77)
Bridging*	29.18 (8.96)	30.84 (8.93)	34.44 (8.96)
Bonding*	29.04 (9.42)	29.80 (9.37)	31.31 (9.42)
Belonging*	53.83 (13.24)	54.52 (13.18)	54.13 (13.24)
Social Connectedness*	79.03 (19.49)	80.38 (19.38)	84.16 (19.47)
Loneliness*	17.22 (5.30)	17.01 (5.26)	16.89 (5.28)
Friends	341.58 (345.43)	444.98 (477.28)	486.65 (526.85)
Hours**	1–2 hours	1–2 hours	2–3 hours

Note:

*Means are adjusted for covariates (see text for details).

**Median self-reported hours spent on Facebook on a typical weekend.

When re-evaluating the *Bridging* subscale of *Social Capital*, although both *Friends* and *Hours* were identified as significant covariates (*F*[1,516] = 15.21, *p* < .001, *η*_*p*_^*2*^ = .03; *F*[1,516] = 6.03, *p* = .014, *η*_*p*_^*2*^ = .01, respectively), the main effect of *Cluster* remained significant (*F*[2,516] = 13.81, *p* < .001, *η*_*p*_^*2*^ = .05). Here, a similar (though not identical) pattern of differences emerged: while the *Interactive* cluster still scored significantly higher (34.44 [8.96]) than both the *Reactive* (30.84 [8.93]; *p* = .001, *d* = .40) and *Passive* clusters (29.18 [8.96]; *p* < .001, *d* = .59), there was no longer any significant difference between the *Reactive* and *Passive* clusters (*p* = .206, *d* = .19). For the Bonding subscale of *Social Capital*, *Friends* again served as a significant covariate (*F*[1,516] = 5.27, *p* = .022, *η*_*p*_^*2*^ = .01) while *Hours* did not (*F*[1,516] = 3.71, *p* = .055, *η*_*p*_^*2*^ < .01). More importantly, the addition of these factors rendered the main effect of *Cluster* non-significant (*F*[2,516] = 2.31, *p* = .100, *η*_*p*_^*2*^ < .01). Similarly, *Friends* was again a significant covariate when re-evaluating the influence of cluster membership on *Social Connectedness* (*F*[1,516] = 8.78, *p* = .003, *η*_*p*_^*2*^ = .02) while *Hours* was not (*F*[1,516] = 2.04, *p* = .154, *η*_*p*_^*2*^ < .01), and the main effect of *Cluster* no longer remained significant with the addition of these covariates (*F*[2,516] = 2.83, *p* = .060, *η*_*p*_^*2*^ = .01). For *Sense of Belonging*, *Hours* was identified as a significant covariate (*F*[1,516] = 5.24, *p* = .023, *η*_*p*_^*2*^ = .01) but *Friends* was not (*F*[1,516] = 2.25, *p* = .134, *η*_*p*_^*2*^ < .01), and the main effect of *Cluster* remained non-significant (*F*[2,516] = .13, *p* = .876, *η*_*p*_^*2*^ = .001). For *Lonelines*s, neither *Friends* (*F*[1,516] = .1.53, *p* = .217, *η*_*p*_^*2*^ < .01) nor *Hours* were identified as significant covariates (*F*[2,516] = .17, *p* = .241, *η*_*p*_^*2*^ < .01) and the non-significant effect of *Cluster* remained (*F*[2,516] = .17, *p* = .842, *η*_*p*_^*2*^ = .001). Refer to Fig 2 for response patterns for each cluster after the addition of the *Friends* and *Hours* covariates.

## Discussion

The present study set out to develop a computerised task capable of delineating among distinct styles of usage on a mock social networking site (SNS), and evaluated its utility for future research into the psychosocial outcomes of different usage styles that might reconcile some of the discrepant findings in this field. Specifically, we designed the Social Networking Site Behaviour Task (SNSBT) as a tool with which researchers can evaluate the claim that interactive SNS usage (content sharing) will lead to more positive psychosocial outcomes than reactive (content liking) and passive usage (content scrolling). Data acquired online from this sample revealed that behaviour on the SNSBT can indeed be classified reliably into these three distinct styles of usage. Comparisons among these styles revealed that individuals exhibiting the strongest tendency for interactive usage reported stronger feelings of social connectedness and scored higher on both sub-dimensions of social capital relative to those expressing more reactive or passive styles. Importantly, however, those who showed the greatest amount of interactive use on the SNSBT also reported the greatest number of Facebook friends and the most amount of time spent on SNS compared with the other two styles. When comparisons among usage styles were corrected for these factors, only the difference between the bridging sub-dimension of social capital remained. We now discuss the implications of these findings for future research in the field of cyberpsychology.

Disparate findings emerging from research into the psychosocial outcomes of SNS usage are attributed frequently to an active-passive dichotomy of behaviour (see reviews by [5, 24]). More recently, however, scholars have questioned the usefulness of such a crude binary distinction, arguing instead for more nuanced classifications that capture with greater accuracy the multi-dimensional nature of behaviour on SNS [8]. For example, unlike the broad definition of active use that includes not only commenting and sending messages but also content liking [24], other scholars differentiate between two-way “truly” interactive (e.g., a continuous message exchange) and two-way reactive usage (e.g., content liking; [33]). Similarly, the interpersonal-connection-behaviours framework [20] makes a distinction between connection- and non-connection-promoting active behaviours on SNS; the former involves interactions whereby users show responsiveness and care towards one another’s needs, while the latter encompasses active usage that fails to contribute to interpersonal connection. Behaviour on the SNSBT provides the first empirical data in support of these finer differentiations: Individuals were dissociated according to their tendency for passive, *re*active, or *inter*active usage, the last of these expressed through content sharing that exposed users to bidirectional exchanges through the feedback they received. These data-driven classifications of usage on the SNSBT also aligned with users’ self-reported tendencies on SNS: whilst all participants perceived their usage of social media to be primarily passive, aligning with previous findings [51], subjective estimates of active usage were greater in individuals with higher proportions of Share responses compared with Like and Next responses. However, responses of the SNSBT were more internally reliable than individuals’ subjective reports. As such, this computerised task provides researchers with a reliable proxy measure of behaviour on SNS that may overcome the limitations of self-report data [5, 25].

Individuals exhibiting more interactive usage reported stronger feelings of connectedness and social capital compared with those who showed more reactive or passive behaviour, and those with a more reactive style of usage reported feeling more connected than those expressing a tendency for passive usage. It is important to stress that these results of comparisons between usage styles on subjective ratings of psychosocial variables provides only a measure of the potential utility of this novel task for future research; they provide no indication of the *outcomes* of SNSBT usage styles. To measure the outcomes of different usage styles on this task, future studies should employ the experimental designs used elsewhere; for example, they might measure changes in users’ mood [52, 53] or subjective well-being [22, 54] before and after specific types of engagement on this task. Alternatively, to capture the outcomes of spontaneous usage styles, future research might employ real-time ecological measurements of psychosocial variables *during* engagement with the SNSBT (see [13].

Perhaps more importantly, our results also revealed that while psychosocial variables differ among individuals according to their response tendencies on the SNSBT, this was often accounted for by the number of friends that participants reported having on Facebook and the amount of time they spent on the platform. This converges with other studies showing that the number of friends and time spent on SNS are associated strongly with social satisfaction and well-being (e.g., [10, 39, 40]; but see [55]). In this light, our findings indicate that usage style operates in concert with, or even emerges as a function of, online network size and time spent on SNS. Indeed, psychological theories of friendship (e.g., [56, 57]) posit that while social networks provide an opportunity for emotional closeness and social support, the degree to which an individual experiences this is dependent upon the intensity and intimacy of relationships they maintain with their network. Larger online social networks reported by interactive users will likely enable them to have more meaningful (bidirectional) exchanges with a wider network of close and casual friends, allowing them to exercise their preferred style more frequently and benefit from it maximally.

As with all experimental measures, the experimental control afforded by the SNSBT as implemented in the current study offers only a limited insight into the complex and multi-dimensional range of behaviours shown on SNS. However, this limitation opens up exciting avenues of research for the field of cyberpsychology. First, like the majority of earlier studies that have investigated differential associations between active and passive usage and psychosocial factors, our experimental task resembles a hybrid of the Facebook and Instagram platforms. As such, it (currently) measures users’ engagement with public image-based content only, not private text-based interactions. Since image-based platforms (e.g., Instagram) appear to have different influences on our well-being compared with text-based alternatives [58], future studies should consider adapting the SNSBT to capture usage styles across alternative platforms. Second, participants in our study received only positive feedback after sharing content with their network. Future work might manipulate this to evaluate the notion that increased well-being from (inter-)active SNS usage is underpinned by positive affective experiences brought about by commenting and chatting [59]. In other words, does negative feedback from posting and sharing content serve to undermine the potential benefits of interactive usage? Similarly, participants in the present study viewed and responded only to positively valenced images of non-social landscapes posted ostensibly by anonymous members of an online network. Although these procedural decisions were made intentionally to avoid multiple confounding factors on participants’ behaviour, the SNSBT presents a tool with which to investigate the impact of more social material on styles of usage. For example, future studies might use different categories of stimuli to build upon research that has explored interactions between usage styles and type of content (e.g., [60]).

Finally, although the current instantiation of the SNSBT incorporated some characteristics of real SNS platforms (i.e. content scrolling, liking, sharing), it did not expose users to other important design features that have been shown to cue habitual social media usage patterns. For example, the hybrid layout of the mock platform differed from either Facebook or Instagram, thereby removing contextual cues that might activate stimulus-response associations that drive particular usage styles (e.g., within-platform chat sounds). Similarly, the non-emotional images used in the current version of the task differ considerably from the unexpected and often emotional content through which users scroll on real SNS platforms, which are considered to be more rewarding and capable of triggering habitual behaviours acquired through reinforcement learning [61–63]. As such, the dissociable styles of usage that we have identified with the SNSBT may not perfectly reflect users’ real-world habits on SNS platforms. However, the ease with which the layout, interface and content of the SNSBT can be modified presents a tool with which these potential drivers of social media habit formation can be investigated experimentally.

## Conclusion

We have developed a simple, publicly available computerised task that provides a new method with which to objectively quantify different styles of behaviour on social networking sites (SNS). The versatility of this task enhances its utility for future research in the field of cyberpsychology; it can be adapted easily to measure usage styles across multiple SNS platforms, to assess content and design features of SNS that might influence discrete usage styles, and perform experimental investigations into the influence of different SNS usage styles on psychosocial outcomes.

## Supporting information

S1 File Exploratory factor analysis of the “passive and active social media use” questionnaire. This presents the inter-item correlation matrix, factor loadings and component plot, which reveal that a two-factor solution is optimal for responses to the Social Media Usage questionnaire [34].
(DOCX)

## References

[1] Tankovska. Number of social network users worldwide from 2017 to 2025 [Internet]. Vol. 44, Statista2. 2020 [cited 2022 Apr 4]. p. 1. Available from: https://www.statista.com/statistics/278414/number-of-worldwide-social-network-users/

[2] Appel M, Marker C, Gnambs T. Are social media ruining our lives? A review of meta-analytic evidence. Rev Gen Psychol. 2020;24(1):60–74.

[3] Huang C. A meta-analysis of the problematic social media use and mental health. Int J Soc Psychiatry. 2022;68(1):12–33. doi: 10.1177/0020764020978434 33295241

[4] Orben A. Teenagers, screens and social media: a narrative review of reviews and key studies. Soc Psychiatry Psychiatr Epidemiol [Internet]. 2020;55(4):407–14. Available from: doi: 10.1007/s00127-019-01825-4 31925481

[5] Kross E, Verduyn P, Sheppes G, Costello CK, Jonides J, Ybarra O. Social media and well-being: Pitfalls, progress, and next steps. Trends Cogn Sci [Internet]. 2021;25(1):55–66. Available from: doi: 10.1016/j.tics.2020.10.005 33187873

[6] Ryan T, Allen KA, Gray DLL, McInerney DM. How social are social media? A review of online social behaviour and connectedness. J Relationships Res. 2017;8:1–8.

[7] Schønning V, Hjetland GJ, Aarø LE, Skogen JC. Social media use and mental health and well-being among adolescents–A scoping review. Front Psychol. 2020;11(August):1–15. 32922333 10.3389/fpsyg.2020.01949 PMC7457037

[8] Valkenburg PM, van Driel II, Beyens I. The associations of active and passive social media use with well-being: A critical scoping review. New Media Soc. 2022;24(2):530–49.

[9] große Deters F, Mehl MR. Does posting Facebook status updates increase or decrease loneliness? An online social networking experiment. Soc Psychol Personal Sci. 2013;4(5):579–86. 10.1177/1948550612469233 PMC3820167 24224070

[10] Ellison NB, Steinfield C, Lampe C. The benefits of facebook “friends:” Social capital and college students’ use of online social network sites. J Comput Commun. 2007;12(4):1143–68.

[11] Sheldon P. Profiling the non-users: Examination of life-position indicators, sensation seeking, shyness, and loneliness among users and non-users of social network sites. Comput Human Behav [Internet]. 2012;28(5):1960–5. Available from: 10.1016/j.chb.2012.05.016

[12] Sinclair TJ, Grieve R. Facebook as a source of social connectedness in older adults. Comput Human Behav [Internet]. 2017;66:363–9. Available from: 10.1016/j.chb.2016.10.003

[13] Kross E, Verduyn P, Demiralp E, Park J, Lee DS, Lin N, et al. Facebook use predicts declines in subjective well-being in young adults. PLoS One. 2013;8(8):1–6. doi: 10.1371/journal.pone.0069841 23967061 PMC3743827

[14] Shakya HB, Christakis NA. Association of Facebook use with compromised well-being: A longitudinal study. Am J Epidemiol. 2017;185(3):203–11. doi: 10.1093/aje/kww189 28093386

[15] Stepanikova I, Nie NH, He X. Time on the Internet at home, loneliness, and life satisfaction: Evidence from panel time-diary data. Comput Human Behav [Internet]. 2010;26(3):329–38. Available from: 10.1016/j.chb.2009.11.002

[16] Griffioen N, van Rooij M, Lichtwarck-Aschoff A, Granic I. Toward improved methods in social media research. Technol Mind, Behav. 2020;1(1).

[17] Orben A, Przybylski AK. Screens, teens, and psychological well-being: Evidence from three time-use-diary studies. Psychol Sci. 2019;30(5):682–96. doi: 10.1177/0956797619830329 30939250 PMC6512056

[18] Parry DA, Davidson BI, Sewall CJR, Fisher JT, Mieczkowski H, Quintana DS. A systematic review and meta-analysis of discrepancies between logged and self-reported digital media use. Vol. 5, Nature Human Behaviour. 2021. 1535–1547 p. doi: 10.1038/s41562-021-01117-5 34002052

[19] Vuorre M, Orben A, Przybylski AK. There is no evidence that associations between adolescents’ digital technology engagement and mental health problems have increased. Clin Psychol Sci. 2021; 10.1177/2167702621994549 PMC7614449 37082461

[20] Clark JL, Algoe SB, Green MC. Social network sites and well-being: The role of social connection. Curr Dir Psychol Sci. 2018;27(1):32–7.

[21] Hall JA. When is social media use social interaction? Defining mediated social interaction. New Media Soc. 2018;20(1):162–79.

[22] Verduyn P, Lee DS, Park J, Shablack H, Orvell A, Bayer J, et al. Passive facebook usage undermines affective well-being: Experimental and longitudinal evidence. J Exp Psychol Gen. 2015;144(2):480–8. doi: 10.1037/xge0000057 25706656

[23] Wang JL, Gaskin J, Rost DH, Gentile DA. The reciprocal relationship between passive social networking site (SNS) usage and users’ subjective well-being. Soc Sci Comput Rev. 2018;36(5):511–22.

[24] Verduyn P, Ybarra O, Résibois M, Jonides J, Kross E. Do social network sites enhance or undermine subjective well-being? A critical review. Soc Issues Policy Rev. 2017;11(1):274–302.

[25] Trifiro BM, Gerson J. Social media usage patterns: Research note regarding the lack of universal validated measures for active and passive use. Soc Media + Soc. 2019;5(2):205630511984874.

[26] Johannes N, Nguyen T, Weinstein N, Przybylski AK. Supplemental material for objective, subjective, and accurate reporting of social media use: No evidence that daily social media use correlates with personality traits, motivational states, or well-being. Technol Mind, Behav. 2021;2(2).

[27] Andrews S, Ellis DA, Shaw H, Piwek L. Beyond self-report: Tools to compare estimated and real-world smartphone use. PLoS One. 2015;10(10):1–9. doi: 10.1371/journal.pone.0139004 26509895 PMC4625000

[28] Ellis DA, Davidson BI, Shaw H, Geyer K. Do smartphone usage scales predict behavior? Int J Hum Comput Stud [Internet]. 2019;130(May):86–92. Available from: 10.1016/j.ijhcs.2019.05.004

[29] Ernala SK, Burke M, Leavitt A, Ellison NB. How well do people report time spent on Facebook? 2020;1–14.

[30] Sewall CJR, Bear TM, Merranko J, Rosen D. How psychosocial well-being and usage amount predict inaccuracies in retrospective estimates of digital technology use. Mob Media Commun. 2020;8(3):379–99.

[31] Kaye LK, Orben A, Ellis DA, Hunter SC, Houghton S. The conceptual and methodological mayhem of “screen time.” Int J Environ Res Public Health. 2020;17(10). 10.3390/ijerph17103661 PMC7277381 32456054

[32] Junco R. Comparing actual and self-reported measures of Facebook use. Comput Human Behav [Internet]. 2013;29(3):626–31. Available from: 10.1016/j.chb.2012.11.007

[33] Meier A, Reinecke L. Computer-mediated communication, social media, and mental health: A conceptual and empirical meta-review. Communic Res. 2020;1–28.

[34] Escobar-Viera CG, Shensa A, Bowman ND, Sidani JE, Knight J, James AE, et al. Passive and active social media use and depressive symptoms among United States adults. Cyberpsychology, Behav Soc Netw. 2018;21(7):437–43. doi: 10.1089/cyber.2017.0668 29995530

[35] Hagerty BMK, Patusky K. Developing a measure of sense of belonging. Nurs Res. 1995;44(1):9–13. 7862549

[36] Lee RM, Robbins SB. Measuring Belongingness: The Social Connectedness and the Social Assurance Scales. J Couns Psychol. 1995;42(2):232–41.

[37] Williams D. On and off the ’net: Scales for social capital in an online era. J Comput Commun. 2006;11(2):593–628.

[38] Dunbar RIM. Do online social media cut through the constraints that limit the size of offline social networks? R Soc Open Sci. 2016;3(1). doi: 10.1098/rsos.150292 26909163 PMC4736918

[39] Sutcliffe AG, Binder JF, Dunbar RIM. Activity in social media and intimacy in social relationships. Comput Human Behav [Internet]. 2018;85:227–35. Available from: 10.1016/j.chb.2018.03.050

[40] Nabi RL, Prestin A, So J. Facebook friends with (health) benefits? Exploring social network site use and perceptions of social support, stress, and well-being. Cyberpsychology, Behav Soc Netw. 2013;16(10):721–7. 10.1089/cyber.2012.0521 23790356

[41] Peer E, Brandimarte L, Samat S, Acquisti A. Beyond the Turk: Alternative platforms for crowdsourcing behavioral research. J Exp Soc Psychol [Internet]. 2017;70:153–63. Available from: 10.1016/j.jesp.2017.01.006

[42] Champely S, Ekstrom C, Dalgaard P, Gill J, Weibelzahl S, Anandkumar A, et al. Basic Functions for power analysis description. 2020;1–22.

[43] Peirce J, Gray JR, Simpson S, MacAskill M, Höchenberger R, Sogo H, et al. PsychoPy2: Experiments in behavior made easy. Behav Res Methods. 2019;51(1):195–203. doi: 10.3758/s13428-018-01193-y 30734206 PMC6420413

[44] Marchewka A, Żurawski Ł, Jednoróg K, Grabowska A. The Nencki Affective Picture System (NAPS): Introduction to a novel, standardized, wide-range, high-quality, realistic picture database. Behav Res Methods. 2014;46(2):596–610. doi: 10.3758/s13428-013-0379-1 23996831 PMC4030128

[45] Riegel M, Żurawski Ł, Wierzba M, Moslehi A, Klocek Ł, Horvat M, et al. Characterization of the Nencki Affective Picture System by discrete emotional categories (NAPS BE). Behav Res Methods. 2016;48(2):600–12. doi: 10.3758/s13428-015-0620-1 26205422 PMC4891391

[46] Pempek TA, Yermolayeva YA, Calvert SL. College students’ social networking experiences on Facebook. J Appl Dev Psychol [Internet]. 2009;30(3):227–38. Available from: 10.1016/j.appdev.2008.12.010

[47] Hays RD, Dimatteo MR. A short-form measure of loneliness. J Pers Assess. 1987;51(1):69–81. doi: 10.1207/s15327752jpa5101_6 3572711

[48] Everitt. Cluster Analysis 5th Edition. 2013. 340–363 p.

[49] Clatworthy J, Buick D, Hankins M, Weinman J, Horne R. The use and reporting of cluster analysis in health psychology: A review. Br J Health Psychol. 2005;10(3):329–58. doi: 10.1348/135910705X25697 16238852

[50] Lakens D. Calculating and reporting effect sizes to facilitate cumulative science: A practical primer for t-tests and ANOVAs. Front Psychol. 2013;4(NOV):1–12. doi: 10.3389/fpsyg.2013.00863 24324449 PMC3840331

[51] Gerson J, Plagnol AC, Corr PJ. Passive and Active Facebook Use Measure (PAUM): Validation and relationship to the Reinforcement Sensitivity Theory. Pers Individ Dif [Internet]. 2017;117:81–90. Available from: 10.1016/j.paid.2017.05.034

[52] Mosquera R, Odunowo M, McNamara T, Guo X, Petrie R. The economic effects of Facebook. Exp Econ [Internet]. 2020;23(2):575–602. Available from: 10.1007/s10683-019-09625-y

[53] Sagioglou C, Greitemeyer T. Facebook’s emotional consequences: Why Facebook causes a decrease in mood and why people still use it. Comput Human Behav. 2014;35:359–63.

[54] Allcott H, Braghieri L, Eichmeyer S, Gentzkow M. The welfare effects of social media†. Am Econ Rev. 2020;110(3):629–76.

[55] Kelly Y, Zilanawala A, Booker C, Sacker A. Social media use and adolescent mental health: Findings from the UK Millennium Cohort Study. EClinicalMedicine [Internet]. 2018;6:59–68. Available from: doi: 10.1016/j.eclinm.2018.12.005 31193561 PMC6537508

[56] Dunbar RIM. The anatomy of friendship. Trends Cogn Sci [Internet]. 2018;22(1):32–51. Available from: doi: 10.1016/j.tics.2017.10.004 29273112

[57] Oswald DL, Clark EM, Kelly CM. Friendship maintenance: An analysis of individual and dyad behaviors. J Soc Clin Psychol. 2004;23(3):413–41.

[58] Pittman M, Reich B. Social media and loneliness: Why an Instagram picture may be worth more than a thousand Twitter words. Comput Human Behav [Internet]. 2016;62:155–67. Available from: 10.1016/j.chb.2016.03.084

[59] Weinstein E. The social media see-saw: Positive and negative influences on adolescents’ affective well-being. New Media Soc. 2018;20(10):3597–623.

[60] Schreurs L, Vandenbosch L. The development and validation of measurement instruments to address interactions with positive social media content. Media Psychol [Internet]. 2021;25(2):262–89. Available from: 10.1080/15213269.2021.1925561

[61] Anderson IA, Wood W. Habits and the electronic herd: The psychology behind social media’s successes and failures. Consum Psychol Rev. 2021;4(1):83–99.

[62] Bayer JB, Anderson IA, Tokunaga RS. Building and breaking social media habits. Curr Opin Psychol [Internet]. 2022;45:101303. Available from: doi: 10.1016/j.copsyc.2022.101303 35255413

[63] Lindström B, Bellander M, Schultner DT, Chang A, Tobler PN, Amodio DM. A computational reward learning account of social media engagement. Nat Commun [Internet]. 2021;12(1):1–10. Available from: 10.1038/s41467-020-19607-x 33637702 PMC7910435

