# Supplementary material for: It’s not what you do, it’s the way that you do it: An experimental task delineates among passive, reactive and interactive styles of behaviour on social networking sites
Source: PLoS One. 2022 Dec 7;17(12):e0276765. doi: 10.1371/journal.pone.0276765 (PMC9728879; doi:10.1371/journal.pone.0276765)

**Supporting Information**

**S1. Exploratory Factor Analysis of the “Passive and Active Social Media Use” questionnaire**

A principal component analysis (PCA) was conducted to assess the factor structure of the Passive and Active Social Media Use questionnaire adapted from Escobar-Viera et al. (2018). Inter-correlations were significant between all nine items of the questionnaire (see Table 1), and both the Kaiser-Meyer-Olkin measure (.81) and Bartlett’s test of sphericity (χ2 [36] = 1564.21, *p* < .001) suggested factorability was appropriate. PCA revealed a two-factor structure for all nine items, with factor 1 (six items, Q4-9) explaining 31.66% of variance and factor 2 (three items, Q1-3) explaining 25.66% of the variance. Given that we replicated the same number of factors as Escobar-Viera et al. (2018), we retained their original factor labels of “Active” and “Passive” social media usage. The SPSS statistical output is appended below.

**Table 1.** *Correlation matrix showing inter-correlations of items from the adapted Passive and Active Social Media Use questionnaire.*

| **Variable** | **1.** | **2.** | **3.** | **4.** | **5.** | **6.** | **7.** | **8.** | **9.** |
| --- | --- | --- | --- | --- | --- | --- | --- | --- | --- |
| 1. I often read discussions when using Facebook. | — |  |  |  |  |  |  |  |  |
| 2. I often read comments/reviews when using Facebook. | 0.71* | — |  |  |  |  |  |  |  |
| 3. I often watch videos or view pictures when using Facebook. | 0.39* | 0.41* | — |  |  |  |  |  |  |
| 4. I often ‘like’ others’ content when using Facebook. | 0.33* | 0.32* | 0.38* | — |  |  |  |  |  |
| 5. I often ‘share’ others’ content when using Facebook. | 0.21* | 0.20* | 0.18* | 0.54* | — |  |  |  |  |
| 6. I often vote on polls when using Facebook. | 0.27* | 0.29* | 0.27* | 0.33* | 0.36* | — |  |  |  |
| 7. I often comment on someone else’s content when using Facebook. | 0.36* | 0.36* | 0.23* | 0.47* | 0.52* | 0.40* | — |  |  |
| 8. I often respond to someone when they comment on my content on Facebook. | 0.27* | 0.34* | 0.30* | 0.42* | 0.22* | 0.27* | 0.43* | — |  |
| 9. I often share my own content on Facebook to receive likes and/or comments. | 0.24* | 0.21* | 0.19* | 0.39* | 0.52* | 0.30* | 0.56* | 0.33* | — |

**Note:** * *p* < .001.

| **Total Variance Explained** | | | | | | | | | |
| --- | --- | --- | --- | --- | --- | --- | --- | --- | --- |
| Component | Initial Eigenvalues | | | Extraction Sums of Squared Loadings | | | Rotation Sums of Squared Loadings | | |
|  | Total | % of Variance | Cumulative % | Total | % of Variance | Cumulative % | Total | % of Variance | Cumulative % |
| 1 | 3.802 | 42.244 | 42.244 | 3.802 | 42.244 | 42.244 | 2.849 | 31.656 | 31.656 |
| 2 | 1.356 | 15.068 | 57.312 | 1.356 | 15.068 | 57.312 | 2.309 | 25.656 | 57.312 |
| 3 | .786 | 8.729 | 66.041 |  |  |  |  |  |  |
| 4 | .752 | 8.351 | 74.392 |  |  |  |  |  |  |
| 5 | .700 | 7.781 | 82.173 |  |  |  |  |  |  |
| 6 | .569 | 6.318 | 88.491 |  |  |  |  |  |  |
| 7 | .395 | 4.391 | 92.882 |  |  |  |  |  |  |
| 8 | .363 | 4.029 | 96.911 |  |  |  |  |  |  |
| 9 | .278 | 3.089 | 100.000 |  |  |  |  |  |  |
| Extraction Method: Principal Component Analysis. | | | | | | | | | |


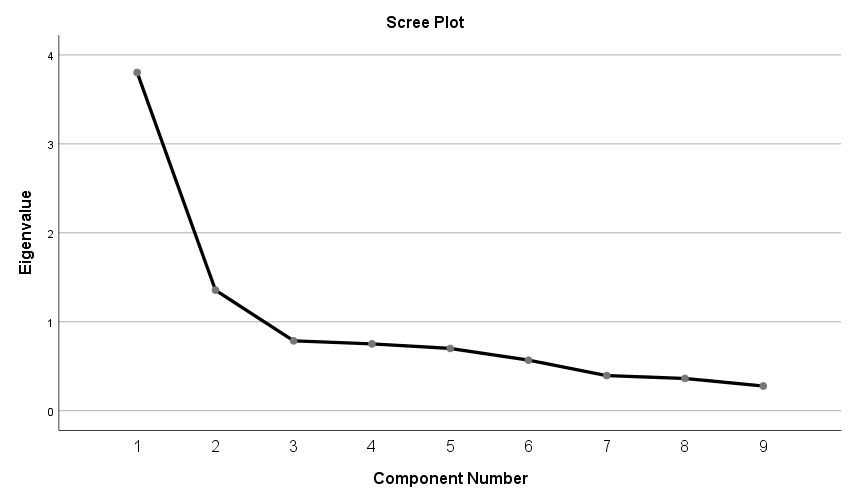


| **Rotated Component Matrix^a^** | | |
| --- | --- | --- |
|  | Component | |
|  | 1 | 2 |
| SMUEscobarQs_5 | .816 |  |
| SMUEscobarQs_9 | .778 |  |
| SMUEscobarQs_7 | .763 |  |
| SMUEscobarQs_4 | .670 |  |
| SMUEscobarQs_6 | .512 |  |
| SMUEscobarQs_8 | .456 |  |
| SMUEscobarQs_2 |  | .861 |
| SMUEscobarQs_1 |  | .836 |
| SMUEscobarQs_3 |  | .663 |
| Extraction Method: Principal Component Analysis.  Rotation Method: Varimax with Kaiser Normalization.^a^ | | |
| a. Rotation converged in 3 iterations. | | |


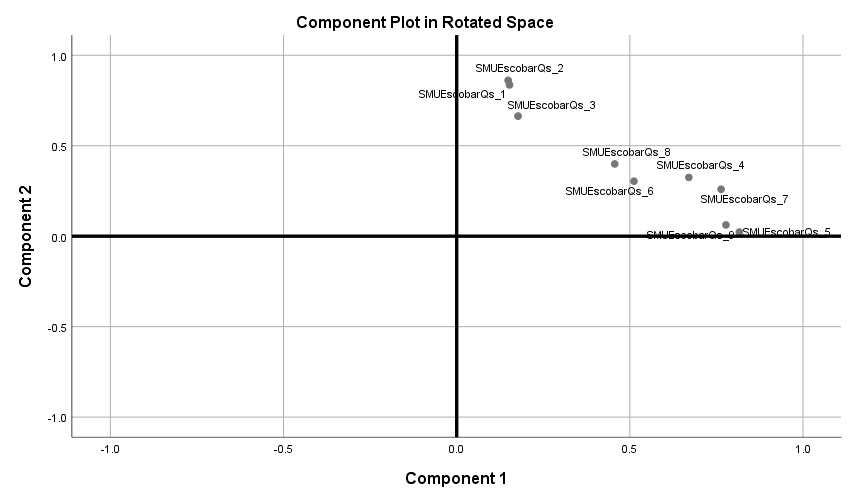

Supplement: S1 File — This presents the inter-item correlation matrix, factor loadings and component plot, which reveal that a two-factor solution is optimal for responses to the Social Media Usage questionnaire [34]. (DOCX) [file pone.0276765.s001.docx]
